# The relationship between two different measures of osteoarthritis bone pathology, bone marrow lesions and 3D bone shape: data from the Osteoarthritis Initiative

**DOI:** 10.1016/j.joca.2018.06.011

**Published:** 2018-10

**Authors:** B. Dube, M.A. Bowes, E.M.A. Hensor, A. Barr, S.R. Kingsbury, P.G. Conaghan

**Affiliations:** † Leeds Institute of Rheumatic and Musculoskeletal Medicine, University of Leeds and NIHR Leeds Biomedical Research Centre, Leeds, UK; ‡ Imorphics Ltd, Manchester, UK

**Keywords:** Bone marrow lesions, Bone shape, Responsiveness, Magnetic resonance imaging, Osteoarthritis

## Abstract

**Objective:**

Bone shape and bone marrow lesions (BMLs) represent different features of Magnetic resonance imaging (MRI)-detected subchondral pathology in osteoarthritis (OA). The aim of this study was to determine how these features are related and how they change in OA progression.

**Methods:**

600 participants from the Osteoarthritis Initiative (OAI) FNIH Biomarkers Initiative were included, having Kellgren–Lawrence grade 1–3, at baseline and MRI data at baseline and 24 months. The associations between 3D quantitative bone shape vectors and presence of (MRI Osteoarthritis Knee Score) MOAKS semi-quantitative BMLs (total BML size ≥1) were analysed for femurs and tibias using linear regression. Responsiveness over 24 months was calculated for both features in four pre-defined progression groups and reported as standardised response means (SRMs). Multilevel models investigated the longitudinal relationship between change in BML size and change in bone shape.

**Results:**

Mean age was 61.5, 59% female and mean body mass index (BMI) 30.7. Correlation between baseline femur vector and BML was *r* = 0.28, *P* < 0.001. The presence of BMLs was associated with higher bone shape vector; coefficient (95% CI) 0.75 (0.54, 0.96) and 0.57 (0.38, 0.77) for femur and tibia respectively, both *P* < 0.001. After covariate adjustment, only the femur remained significant [coefficient 0.49, (95% CI 0.30, 0.68)]. Longitudinally bone vector demonstrated more responsiveness to change than BMLs (SRM 0.89 vs 0.13) while multilevel models revealed that increase in BML size was related to a more positive bone shape vector (representing worsening OA).

**Conclusion:**

There is a relationship between bone shape and BMLs, with prevalence of BMLs associated with increasing OA bone shape. Bone shape demonstrated greater responsiveness than semi-quantitative BMLs.

## Introduction

Magnetic resonance imaging (MRI) has provided insights into the development of osteoarthritis (OA) and helped demonstrate the importance of subchondral bone pathology^1^. Bone is important in OA pathogenesis and biomarker development, and bone marrow lesions (BMLs) are one of the most studied of these bone pathologies^2^. BMLs are high signal MRI lesions that have been associated with other pathologies and symptoms^3^, ^4^, ^5^, and their predictive validity has also been reported^4^.

Research on another OA pathological manifestation, change in 3-dimensional bone shape, has emerged. Bone shape which incorporates both spreading of bone and osteophytic changes^6^, ^7^ has shown to be more responsive than current radiographic and standard MRI measures of cartilage for assessing OA progression^8^, predictive of incident radiographic OA^7^, and associated with joint replacement^9^.

While it is appreciated that subchondral bone changes play an important role in OA pathogenesis^2^ the relationship between these two measures (BMLs and 3D bone shape) remains poorly studied. It is important to understand if they represent a single construct or different parts of the OA process, and to further explore their use as imaging biomarkers. The aim of this study was therefore to evaluate the relationship between BMLs and 3D bone shape and their change over time.

## Methods

### Participants

Participants were selected from the Osteoarthritis Initiative (OAI), a longitudinal cohort of 4,796 participants with clinical, radiological, biochemical and other data collected at baseline and annual follow-up visits, available at http://www.oai.ucsf.edu/.The OAI recruited participants with symptomatic and radiographic OA, and also those with no OA but considered at high risk of incident OA. The current study included 600 participants from the Foundation for the NIH Biomarkers Consortium (FNIH) OA, a sub-study aimed at establishing the predictive and concurrent validity and responsiveness of biomarkers for knee OA. More details including inclusion criteria are available at https://oai.epi-ucsf.org/datarelease/FNIH.asp. Four knee categories have been defined in the FNIH: Group 1 (both radiographic and pain progression), Group 2 (radiographic but not pain progression), Group 3 (pain but not radiographic progression) and Group 4 (neither radiographic nor pain progression).

### MR image acquisition and quantitative measures

MRI images were scored for BMLs using the semi-quantitative (SQ) (MRI Osteoarthritis Knee Score) MOAKS system^10^. For each sub-region MOAKS scores three features using an ordinal score for size, number of BMLs and percentage of lesion that is a BML. Quantitative 3D bone shape data was provided by Imorphics (Manchester, UK) using active appearance models applied separately for femur and tibia from automated segmentation of sagittal Double Echo Steady State 3-Tesla images from the OAI. The anatomical regions for derivation of bone shape measures were the whole distal femur and proximal tibia bones^6^, ^7^, ^8^. The training set for the segmentation model, and a separate training set for determining the mean shape of the OA and non-OA bones were independent of the test set. The OA vector for each bone was defined as the line passing through the mean shape for the two populations. This is determined by taking the mean non-OA shape, and the mean OA shape, parameterised using the shape model, and drawing a straight line through the means. The origin of each OA vector is defined as the mean non-OA shape, and distances along the vector are normalised so that +1 represents the mean non-OA shape and −1 the mean OA shape. For ease of interpretation we rescaled the vector using a correction factor of (−1) such that +1 refers to mean OA shape. Reproducibility of MOAKS and shape vector has been reported elsewhere^7^, ^10^.

## Statistical analysis

Statistical analysis was performed using Stata 13.1 software (StataCorp, TX, USA). Bone shape vectors, one each for femur and tibia were compared with corresponding BML regions to match MOAKS scoring. The patella vector (excluded) as it did not directly compare with the patella region on MOAKS. Four BML scores were computed: “total BML size” (computed separately for the femur and tibia by summing the BML size scores in those regions, which combined the six sub-regions in the femur, total possible score = 18 and similarly for the tibia); “total BML number” (summing the number of BMLs in each sub-region); a “maximum BML size” (the highest grade across the femur (ranging 0–3) and similarly for the tibia); and “total BML sub-regions” calculated by summing the total number of sub-regions within the femur and tibia affected by any BML (ranging from 0 to 6).

Baseline correlation between bone measures was assessed using Spearman's correlation. The proportions of participants with shape vector scores outside “healthy limits”, defined as the upper 95^th^ percentile of normal knees (femur shape ≥0.96 on vector scale) were compared between participants having BMLs vs those without. Linear regression was used to evaluate associations between bone shape and presence of BMLs at baseline, adjusting for age, sex, body mass index (BMI), physical activity score (PASE) and Kellgren Lawrence (KL) grade chosen a priori from clinical knowledge. The incidence of BMLs at follow-up was reported descriptively and compared to changes in bone vectors for changes greater than the smallest detectable difference, SDD. SDD for the femur vector was 0.24 units and 0.59 for tibia.

Longitudinal relationships between change in femur vector and change in total BML size were assessed using multilevel linear models, incorporating the effect of time while adjusting for covariates as before. Initially unconditional growth models were assessed for both features. The effect of baseline variables (BML size at baseline) in predicting change in bone vector was modelled by fitting models with baseline total BML size and an interaction term (baseline total BML size × time) to the unconditional growth model for 3D shape. Lastly, BMLs were modelled as time-varying predictors by including both time and BMLs as independent variables adjusting for covariates as before. Level of significance was set at *P* < 0.05.

Longitudinal analyses also explored group-level internal responsiveness using standardised response means (SRMs), and to aid comparison with previous FNIH studies we additionally used the “maximum BML size” and “total BML sub-regions”. SRMs were analysed within each outcome group, since expected changes were assumed homogenous within these groups. Responsiveness was also explored by KL grade.

## Results

### Baseline findings

The mean (SD) age was 61.5 (8.88) years, 59% female with mean (SD) BMI of 30.7 (4.78) and median (IQR) PASE score of 154.5 (102–214). BML prevalence at baseline was 71% for the femur and 41% for tibia (Table I), while 26% had femur vector outside healthy limits. Bone shape vector scores outside healthy limits were more prevalent in participants with BMLs compared to those without (31% vs 14% respectively, Χ^2^ (1df) = 17.50, *P* < 0.001). Moderate positive correlation was seen between femur vector and femur BML total size, *r* (598) = 0.31, *P* < 0.001 while a small positive correlation was seen for the tibia, *r* (598) = 0.16, *P* < 0.001. Analyses with total BML numbers revealed similar associations.

**Table I:** Clinical and radiographic features at baseline and 24 months responsiveness

	Radiographic and pain progression	Radiographic progression only	Pain progression only	No progression
	*N* = 194	*N* = 103	*N* = 103	*N* = 200
Baseline findings				
Age, mean (SD)	62.0 (8.8)	63.1 (8.3)	59.2 (9.1)	61.5 (9.1)
Sex, female *n*, (%)	110 (57)	46 (45)	67 (65)	130 (65)
BMI, mean (SD)	30.7 (4.8)	30.7 (4.7)	31.1 (5.0)	30.5 (4.8)
PASE, median (IQR)	148.5 (102–202)	176.5 (114–246)	156.0 (115–235)	150.0 (89–208)
KL grade *n*, (%)				
1	24 (12.4)	14 (13.6)	13 (12.6)	24 (12)
2	84 (43.4)	47 (45.6)	61 (59.2)	114 (57)
3	86 (44.3)	42 (40.8)	29 (28.2)	62 (31)
Femur shape vector	+0.35 (1.29)	+0.31 (1.21)	−0.04 (1.08)	−0.11 (1.23)
Tibia shape vector	+0.34 (1.25)	+0.35 (1.26)	+0.03 (1.11)	+0.02 (1.17)
Femur BML, present, *n* (%)	155/194 (80)	79/103 (77)	65/103 (63)	124/200 (62)
Tibia BML, present, *n* (%)	103 (53)	53 (51)	32 (31)	60 (30)
Patella BML, present, *n* (%)	143 (74)	65 (63)	68 (66)	141 (71)
**2 year responsiveness, SRM (95%CI)**				
Bone shape vector				
Femur	0.89 (0.72,1.02)	1.02 (0.85,1.20)	0.46 (0.31,0.61)	0.61 (0.49,0.72)
Tibia	0.84 (0.70,0.97)	0.76 (0.56,0.96)	0.26 (0.07,0.43)	0.47 (0.33,0.69)
BMLs				
Femur total BML size	−0.13 (−0.26,0.02)	−0.15 (−0.35,0.07)	−0.31 (−0.51,–0.13)	−0.24 (−0.37,0.13)
Femur total BML number	0.38 (0.26,0.50)	0.20 (−0.02,0.41)	0.17 (−0.04,0.35)	0.27 (0.14,0.38)
Tibia total BML size	0.11 (−0.02,0.26)	0.14 (−0.04,0.31)	−0.04 (−0.23,0.15)	−0.01 (–0.16,0.11)
Tibia total BML number	0.37 (0.24,0.51)	0.31 (0.12,0.51)	0.19 (0.00,0.33)	0.16 (0.02,0.29)
Femur maximum BML size	−0.05 (−0.19,−0.09)	−0.01 (−0.19,0.21)	−0.20 (−0.38,0.01)	−0.11 (−0.25,0.02)
Total BML sub regions	−0.02 (−0.15,0.13)	−0.03 (−0.23,0.15)	−0.13 (−0.32,0.07)	−0.06 (−0.20,0.08)

Linear models revealed an association between presence of a femur BML at baseline and baseline 3D femur vector in both univariable and multivariable models (adjusted coefficient 0.49, 95% CI 0.30, 0.68) (indicative of “increased OA”) in individuals with BMLs at baseline, with a difference equivalent to 0.5 × SD of non-OA knees. For the tibia only univariable models showed an association (Table II). Model diagnostics revealed no departures from normality.

**Table II:** Cross-sectional and longitudinal association between bone shape and BMLs

Univariable models	Coefficient (95% CI)	*P*-value	Multivariable models	Coefficient (95% CI)	*P*-value
**Cross-sectional models**					
Femur BML (present)	0.75 (0.54,0.96)	<0.001*	Femur BML (present)	0.49 (0.30,0.68)	0.03*
			PASE (square root)	−0.02 (−0.05,0.01)	0.10
KL grade (ref = KL1)			Age	−0.01 (−0.01,0.01)	0.83
Grade 2	0.50 (0.21,0.79)	0.001*	BMI	0.03 (0.01,0.05)	<0.001*
Grade 3	1.30 (0.99,1.60)	<0.001*	Gender (ref = female)	−0.97 (−1.15,–0.80)	<0.001*
			KL grade (ref = KL1)		
			KL grade 2 KL grade 3	0.35 (0.08,0.61) 0.94 (0.66,1.22)	0.01* <0.001*
Tibia BML (present)	0.57 (0.38,0.77)	<0.001*	Tibia BML (present)	0.07 (−0.13,0.27)	0.50
			PASE (square root)	−0.01 (−0.04,0.02)	0.38
KL grade (ref = KL1)			Age	−0.01 (−0.01,0.01)	0.86
Grade 2	0.67 (0.39,0.95)	<0.001*	BMI	0.02 (−0.01,0.04)	0.08
Grade 3	1.36 (1.07,1.66)	<0.001*	Gender (ref = female)	0.20 (0.01,0.39)	0.04*
			KL grade (ref = KL1)		
			KL grade 2 KL grade 3	0.62 (0.33,0.90) 1.33 (1.02,1.65)	<0.001* <0.001*
**Multilevel models**					
Unconditional growth	Estimate (standard error)	*P*-value	Multivariate models	Estimate (standard error)	*P*-value
Femur vector intercept	0.12 (0.05)	<0.001*	Femur baseline BML	0.24 (0.03)	<0.001*
Femur Slope	0.11 (0.01)	0.02*	Femur BML Slope	0.01 (0.002)	0.007*
Femur BML intercept	1.37 (0.06)	<0.001*			
Femur BML slope	−0.11 (0.03)	<0.001*			
Tibia vector intercept	0.18 (0.05)	<0.001*	Tibia baseline BML	0.15 (0.04)	<0.001*
Tibia Slope	0.12 (0.01)	<0.001*	Tibia BML Slope	0.01 (0.01)	0.43
Femur BML intercept	0.77 (0.05)	<0.001*			
Femur BML slope	0.04 (0.03)	0.13			

∗ = statistically significant

### Incident BML findings

There were 53 incident cases of femur BMLs over the duration of the study; 21 (40%) of these showed femur vector changes greater than SDD compared to 211/547 (39%) in participants with no incident BMLs. Similar results were seen for the tibia.

### Longitudinal (2-year association)

Univariable growth models revealed that bone shape vector tended to be more positive over time (indicating worsening) while total BML size reduced over time (Table II). When modelled simultaneously to include the effect of an interaction with time, increased baseline femur total BML size was related to more positive (more OA-like) femur vector (Coefficient = 0.24, *P* < 0.001), and an increase in total BML size over time was associated with increase or worsening of the vector over time (Coefficient = 0.01, *P* = 0.007 (Table II). Baseline findings were similar for the tibia but the longitudinal association was not statistically significant (Table II).

Bone vector was more responsive than both SQ total BML size and total BML number scores in all regions over 2 years [femur vector (SRM = 0.89, 95% CI 0.72,1.02) vs femur total BML size (SRM –0.13, 95% CI –0.26,0.02)]. Similar results were found when responsiveness was compared by KL grade (results not shown). Bone vector was also more responsive than the maximum BML size score and the total BML sub-regions (only femur results shown, Table I).

## Discussion

This is the first study to examine the relationship between 3D bone shape and a relatively well studied bone pathology, BMLs. The study investigated their inter-relationship and relative responsiveness as imaging biomarkers. We found a weak positive correlation between bone shape and total BML score in cross-sectional analysis and femoral bone shape was associated with prevalent BMLs. This is plausible since worsening OA status (as measured using bone vector) has been shown to relate to incident radiographic OA and its progression^1^, ^8^ and BMLs have been associated with OA prevalence and progression^3^, ^4^. The relative importance of the femur (over the tibia) may be explained by its larger articulating surface area compared to the tibia. 3D femoral bone vector is also independently associated with incident radiographic OA and total knee replacement^1^, ^9^.

A recent systematic review^1^ concluded that subchondral bone features (including BMLs and bone shape) were independently associated with clinical features such as pain and joint replacement^7^, ^9^. However for both features, most studies reported wide CIs and lower limits of CIs close to 1.0 for ORs and 0 for regression coefficients^1^. There have been fewer studies exploring bone shape, and recently Hunter *et al.* found modest associations between changes in bone shape and pain progression^6^. In our 2 year study, bone vector changes (beyond that of non-OA knees) were seen in individuals with no incident BMLs at follow-up, suggesting bone shape change precedes BML formation^2^.

Similar to our baseline findings, longitudinal analyses found modest associations between bone vector and BMLs. In terms of their relative use as imaging biomarkers in OA clinical trials, this study demonstrated that bone vector is a more responsive measure than SQ assessment of BMLs. There is limited literature comparing OA imaging biomarkers to date. Using the same FNIH cohort, Hunter *et al.* showed that bone shape was associated with radiographic and pain progression longitudinally^6^, while imaging biomarkers of bone (including bone shape and BML measures used in this study) were only weakly associated with OA biochemical biomarkers; however bone shape and BMLs were not directly compared^11^. In the same cohort, Collins *et al.* used SQ imaging biomarkers of OA progression to explore the effect of a combination of joint structures on OA progression and reported that changes in BMLs were not significant predictors of progression in models that already included cartilage, meniscus, and effusion markers^12^. Bone has been shown to be more responsive than radiographic measures of progression such as JSW and also MRI-derived cartilage thickness measures^8^ and other studies with similar follow-up duration reported low responsiveness for BMLs^13^.

There are limitations to this study. Firstly in trying to understand the temporal nature of different bone pathological changes, we only followed participants over a 2 year period. Secondly the study selected participants chosen for the presence or absence of structural/pain progression and may not represent a broader population sample. Definition of change in SQ measures was challenging due to various BML score combinations and there are drawbacks with SQ measures, such as comparing a summed score for BMLs when only one of six sub-regions scores the maximum and the other five score zero. Also, BMLs fluctuate in size over time which may reduce their responsiveness.

This study has suggested a relationship between bone shape and BMLs, provided some evidence for the temporal order of MRI-detected OA bone pathologies and demonstrated the better responsiveness of 3D bone shape over semi-quantitatively assessed BMLs over time periods typical of a clinical trial.

## Author contributions

All authors contributed to the planning and design of this analysis. BD, MB & PC drafted the article and, SK, EH, and AB revised the article. All authors approved the final version for publication.

## Competing interests

Mike Bowes is an employee and shareholder of Imorphics Ltd (a wholly owned subsidiary of Stryker Corp).

Bright Dube, Andrew Barr, Elizabeth Hensor, Sarah Kingsbury, and Philip Conaghan have nothing to disclose.

## Role of the funding source

This study has been part funded by Arthritis Research UK (grant 20800), the Arthritis Research UK Experimental Osteoarthritis Treatment Centre (Ref 20083), the Arthritis Research UK Centre for Sport, Exercise and Osteoarthritis (Ref 20194), and the National Institute for Health Research (NIHR) through the Leeds Biomedical Research Centre. This article/paper/report presents independent research funded in part by the NIHR. The views expressed are those of the authors and not necessarily those of the NHS, the NIHR or the Department of Health.

None of the Sponsors had any involvement in the design, collection, analysis or interpretation of the data and no role in writing or submitting this manuscript for publication.
